# Imaging of chemokine receptor CXCR4 expression in culprit and nonculprit coronary atherosclerotic plaque using motion-corrected [^68^Ga]pentixafor PET/CT

**DOI:** 10.1007/s00259-018-4076-2

**Published:** 2018-07-03

**Authors:** Thorsten Derlin, Daniel G. Sedding, Jochen Dutzmann, Arash Haghikia, Tobias König, L. Christian Napp, Christian Schütze, Nicole Owsianski-Hille, Hans-Jürgen Wester, Saskia Kropf, James T. Thackeray, Jens P. Bankstahl, Lilli Geworski, Tobias L. Ross, Johann Bauersachs, Frank M. Bengel

**Affiliations:** 1 Department of Nuclear Medicine, Hannover Medical School, Carl-Neuberg-Str. 1, 30625 Hannover, Germany; 2 Department of Cardiology and Angiology, Hannover Medical School, Carl-Neuberg-Str. 1, 30625 Hannover, Germany; 3 Department of Radiation Protection and Medical Physics, Hannover Medical School, Carl-Neuberg-Str. 1, 30625 Hannover, Germany; 4 Radiopharmaceutical Chemistry, Technical University of Munich, Munich, Germany; 5 Scintomics GmbH, Fürstenfeldbruck, Germany

**Keywords:** Positron emission tomography, Atherosclerosis, Plaque, CXCR4, Myocardial infarction

## Abstract

**Purpose:**

The chemokine receptor CXCR4 is a promising target for molecular imaging of CXCR4^+^ cell types, e.g. inflammatory cells, in cardiovascular diseases. We speculated that a specific CXCR4 ligand, [^68^Ga]pentixafor, along with novel techniques for motion correction, would facilitate the in vivo characterization of CXCR4 expression in small culprit and nonculprit coronary atherosclerotic lesions after acute myocardial infarction by motion-corrected targeted PET/CT.

**Methods:**

CXCR4 expression was analysed ex vivo in separately obtained arterial wall specimens. [^68^Ga]Pentixafor PET/CT was performed in 37 patients after stent-based reperfusion for a first acute ST-segment elevation myocardial infarction. List-mode PET data were reconstructed to five different datasets using cardiac and/or respiratory gating. Guided by CT for localization, the PET signals of culprit and various groups of nonculprit coronary lesions were analysed and compared.

**Results:**

Ex vivo, CXCR4 was upregulated in atherosclerotic lesions, and mainly colocalized with CD68^+^ inflammatory cells. In vivo, elevated CXCR4 expression was detected in culprit and nonculprit lesions, and the strongest CXCR4 PET signal (median SUV_max_ 1.96; interquartile range, IQR, 1.55–2.31) was observed in culprit coronary artery lesions. Stented nonculprit lesions (median SUV_max_ 1.45, IQR 1.23–1.88; *P* = 0.048) and hot spots in naive remote coronary segments (median SUV_max_ 1.34, IQR 1.23–1.74; *P* = 0.0005) showed significantly lower levels of CXCR4 expression. Dual cardiac/respiratory gating provided the strongest CXCR4 PET signal and the highest lesion detectability.

**Conclusion:**

We demonstrated the basic feasibility of motion-corrected targeted PET/CT imaging of CXCR4 expression in coronary artery lesions, which was triggered by vessel wall inflammation but also by stent-induced injury. This novel methodology may serve as a platform for future diagnostic and therapeutic clinical studies targeting the biology of coronary atherosclerotic plaque.

**Electronic supplementary material:**

The online version of this article (10.1007/s00259-018-4076-2) contains supplementary material, which is available to authorized users.

## Introduction

The C-X-C chemokine receptor 4 (CXCR4) is a transmembrane G-protein-coupled receptor that plays a pivotal role in recruitment of immune and progenitor cells to injured and inflamed tissue via interaction with its ligand CXCL12 [1, 2]. In atherosclerosis, the CXCL12/CXCR4 axis exerts atherogenic, prothrombotic, and plaque-destabilizing effects [3, 4]. CXCR4 is highly expressed by monocytes, differentiated macrophages and lymphocytes migrating into arterial lesions [4–6], and also by platelets [7]. In porcine models of coronary injury, CXCR4^+^ leucocytes have been shown to enter the injured tissue [6]. Furthermore, CXCR4 is also expressed by different cell types including smooth muscle cell progenitors and endothelial progenitor cells which contribute to plaque evolution [4]. Accordingly, CXCR4 may be a useful target for noninvasive molecular imaging, e.g. to determine the degree of inflammation, the likelihood of lesion progression or repair, and the effect of potential targeted therapies in injured atherosclerotic plaques.

To this end, combined PET/CT has emerged as a well-characterized imaging technique to assess plaque biology via mechanisms such as increased metabolism and microcalcification [8–10]. Recently, a promising CXCR4-specific ligand, [^68^Ga]pentixafor [11], has been introduced for clinical molecular imaging of CXCR4 expression. [^68^Ga]Pentixafor identifies myocardial inflammation early after acute myocardial infarction [12, 13], and first both experimental and clinical studies support its use to determine vessel wall CXCR4 expression [14–16]. However, the use of PET/CT for imaging coronary vessels is complicated by the small target size and by blurring from respiratory and cardiac motion. Techniques for motion correction have been proposed to overcome these challenges [17], but have only recently become available for routine clinical application.

Supported by histological verification of the molecular target in plaque specimens, we speculated that CXCR4-targeted PET/CT combined with novel motion-correction techniques may enable in vivo detection of CXCR4-expressing cells in coronary atherosclerotic plaque in the clinical setting. We tested this specific hypothesis in a retrospective analysis of patients who had undergone PET early after reperfusion for acute myocardial infarction, where a spectrum of coronary plaques ranging from culprit injured to nonculprit lesions are readily available as imaging targets.

## Materials and methods

### Ex vivo tissue analysis

For ex vivo validation, cadaveric coronary artery specimens with or without atherosclerotic lesions and carotid plaque specimens from patients with symptomatic or asymptomatic severe carotid stenosis who had undergone carotid endarterectomy were analysed by immunofluorescence microscopy, real-time PCR, immunoblotting and autoradiography for CXCR4 expression and its cellular substrate. A detailed description of the ex vivo techniques is available in the Supplementary material.

### Patients

[^68^Ga]Pentixafor PET/CT scans were performed in 37 patients (median 62.4 years; interquartile range, IQR, 51.8–70.8 years) within 1 week of stent-based reperfusion therapy for a first acute ST-segment elevation (STEMI) myocardial infarction, for clinical assessment of postinfarction myocardial inflammation. Datasets were retrospectively analysed for the presence of uptake within the coronary arteries. Relevant clinical characteristics of the study population are shown in Table 1. No patients with a history of large vessel vasculitis were included in this study. At the time of infarction, ten patients were receiving treatment with β-blockers, nine with angiotensin-converting-enzyme (ACE) inhibitors, nine with angiotensin II receptor antagonists, seven with statins, and six with low-dose acetylsalicylic acid. One patient was receiving treatment with ibuprofen. No patient was treated with other antiinflammatory drugs. Noninvasive imaging had been performed for clinical purposes, i.e. to determine inflammatory burden in the infarct region [12, 13], since suppressed or unrestricted postinfarction inflammation increases the risk of adverse remodelling, may result in impaired cardiac function and heart failure [13], and identifies subjects who require more careful follow up. Patients provided written informed consent before imaging. [^68^Ga]Pentixafor was used clinically according to Section 13.2b of the German Medicinal Products Act. The local institutional review board approved the data analysis, and the need of a formal review was waived. The study complied with the principles of the Declaration of Helsinki.

**Table 1:** Patient characteristics

Parameter	Value
No. of patients	37
Age (years), median (IQR)	62.4 (51.8–70.8)
Gender (male/female), *n*	30/7
Cardiovascular risk profile, *n* (%)	
Arterial hypertension	20 (54)
Hyperlipidaemia	14 (38)
Diabetes mellitus	9 (24)
Smoking	20 (54)
Obesity^a^	8 (22)
Renal Insufficiency^b^	2 (5)
Culprit vessel, *n* (%)^c^	
LAD	24 (63)
LCX	3 (8)
RCA	11 (29)
Time intervals (h), median (IQR)	
Symptoms to intervention	3 (2–12)
Intervention to PET	96 (73–128)
Symptoms to PET	105 (75–133)

*IQR* interquartile range, *LAD* left anterior descending coronary artery, *LCX* left circumflex coronary artery, *RCA* right coronary artery

^a^Body mass index >30 kg/m^2^

^b^Estimated glomerular filtration rate <60 ml/min/1.73 m^2^

^c^38 culprit lesions

### PET/CT imaging

[^68^Ga]Pentixafor was synthesized as previously described [18, 19] using a ^68^Ge/^68^Ga generator (Eckert & Ziegler, Braunschweig, Germany) connected to an automated module (Scintomics, Fürstenfeldbruck, Germany). All studies were conducted using a dedicated PET/CT system (Biograph mCT 128 Flow; Siemens, Knoxville, TN). Patients received an intravenous injection of [^68^Ga]pentixafor (median dose 129 MBq, IQR 107–150 MBq). Imaging began with a low-dose CT scan (120 kV, mA modulated, pitch 1.2, reconstructed axial slice thickness 5.0 mm) for attenuation correction of PET images. List-mode PET was acquired starting 60 min after injection over 30 min, with electrocardiographic and respiratory gating (Anzai AZ733 V system; Anzai Medical Co, Tokyo, Japan). In addition to ungated PET images, list-mode data were resampled to various gated datasets, to correct for motion. Specifically, datasets were created using cardiac [20], amplitude-based respiratory [21, 22], list-mode data-driven respiratory [23, 24], and dual cardiac and respiratory gating [25]. For cardiac gating, eight time bins were created and the end-diastolic bin was used for analysis. For amplitude-based respiratory gating, a duty cycle of 35% was employed that provided balance between image quality and motion rejection [21, 22]. List-mode data-driven gating (MFL, “motion from list-mode”; Siemens, Knoxville, TN) was also performed with a duty cycle of 35%, combined with an optimal respiratory gating algorithm to determine the best amplitude range. For dual respiratory and cardiac gating, a combination of amplitude-based respiratory duty cycles of 35% and cardiac end-diastolic-phase was used [21, 25]. All studies were reconstructed using time-of-flight and point-spread function information combined with an ordered subsets expectation maximization algorithm (TrueX®; Siemens Healthcare).

### PET/CT analysis

Transaxial [^68^Ga]pentixafor PET, CT and fused PET/CT images were analysed using commercial software (*syngo*.via; Siemens Healthcare). Images were analysed by an experienced reader (T.D., with >10 years experience of PET plaque imaging). PET images were read in conjunction with CT images to identify the stented lesion and with coronary angiograms to identify the site of the culprit lesion. First, ungated images were evaluated for the presence of focally increased tracer uptake (higher than background) in the stented culprit lesion. Then, the different gated datasets were likewise evaluated for the presence of focal tracer uptake in the stented culprit lesion. Using the dataset with the highest detection rate (dual-gated images), four groups of atherosclerotic lesions were then evaluated accordingly:

- *Group 1* consisted of the *culprit* lesions, which led to coronary occlusion on angiography and were identified on PET/CT images by CT-based localization of stents placed for reperfusion: 38 lesions were identified in 37 patients, 24 (63%) in the left anterior descending coronary artery (LAD), 11 (29%) in the right coronary artery (RCA), and 3 (8%) in the left circumflex coronary artery (LCX).
- *Group 2* consisted of *nonculprit* lesions which did not lead to coronary occlusion but were stented to treat significant stenosis (at least 50% diameter narrowing of a major coronary artery) in the same session: 12 lesions were identified.
- *Group* 3 consisted of *nonculprit* *nonstented* coronary lesions (<50% diameter narrowing of a major coronary artery) which were identified on PET/CT images as a focal hot spot of CXCR4 upregulation fusing to a coronary artery: 36 lesions were identified in 22 patients.
- *Group 4* consisted of *nonculprit* *nonstented* coronary lesions (<50% diameter narrowing of a major coronary artery), which were identified on PET/CT images as *calcified* lesions in a noninfarct vessel: 37 lesions were identified (one intra-individual control lesion per patient).

All PET images were visually evaluated for the presence of focal radiotracer uptake (higher than background). Additionally, maximum standardized uptake values (SUV_max_) as a measure of signal intensity in target regions were obtained by manually placing an individual circular volume of interest (VOI) around the lesion. Tracer uptake in myocardial tissue was determined using an additional VOI placed in the infarcted area. Mean SUVs were also obtained for thoracic vertebra bone marrow and spleen using VOIs of diameter 2 cm.

### Statistical analysis

Continuous variables are expressed as medians with interquartile ranges (IQR). Categorical variables are presented with absolute and relative frequencies. The D’Agostino-Pearson omnibus normality test was used to confirm that values were normally distributed. Lesion detectability using different gated and ungated reconstructions was compared using Fisher’s exact test. A repeated-measures one-way ANOVA with the Greenhouse-Geisser correction with Dunett’s multiple comparison test was also applied. As retrospective reconstruction of PET data using dual cardiac and respiratory gating could not be performed in three patients (8%) because of missing values, these data were compared with ungated data using a paired *t* test. A one-way ANOVA with Dunett’s multiple comparison test was used to compare tracer uptake between stented culprit lesions, stented nonculprit lesions, nonculprit CXCR4^+^ lesions, and nonculprit calcified control lesions. Multiplicity-adjusted *P* values are reported. Arterial tracer uptake was correlated with time intervals and tracer uptake in organs using Pearson correlation. Ex vivo data among study groups were analysed using the unpaired Student’s *t* test. Statistical significance was considered established for *P* values of less than 0.05. Data were stored and analysed on personal computers using Microsoft Excel 2010. Statistical analysis was performed using GraphPad Prism (version 6.01 for Microsoft Windows; GraphPad Software).

## Results

### Ex vivo CXCR4 is upregulated in atherosclerotic plaque specimens and localizes mainly to CD68^+^ cells

**Fig. 1:**
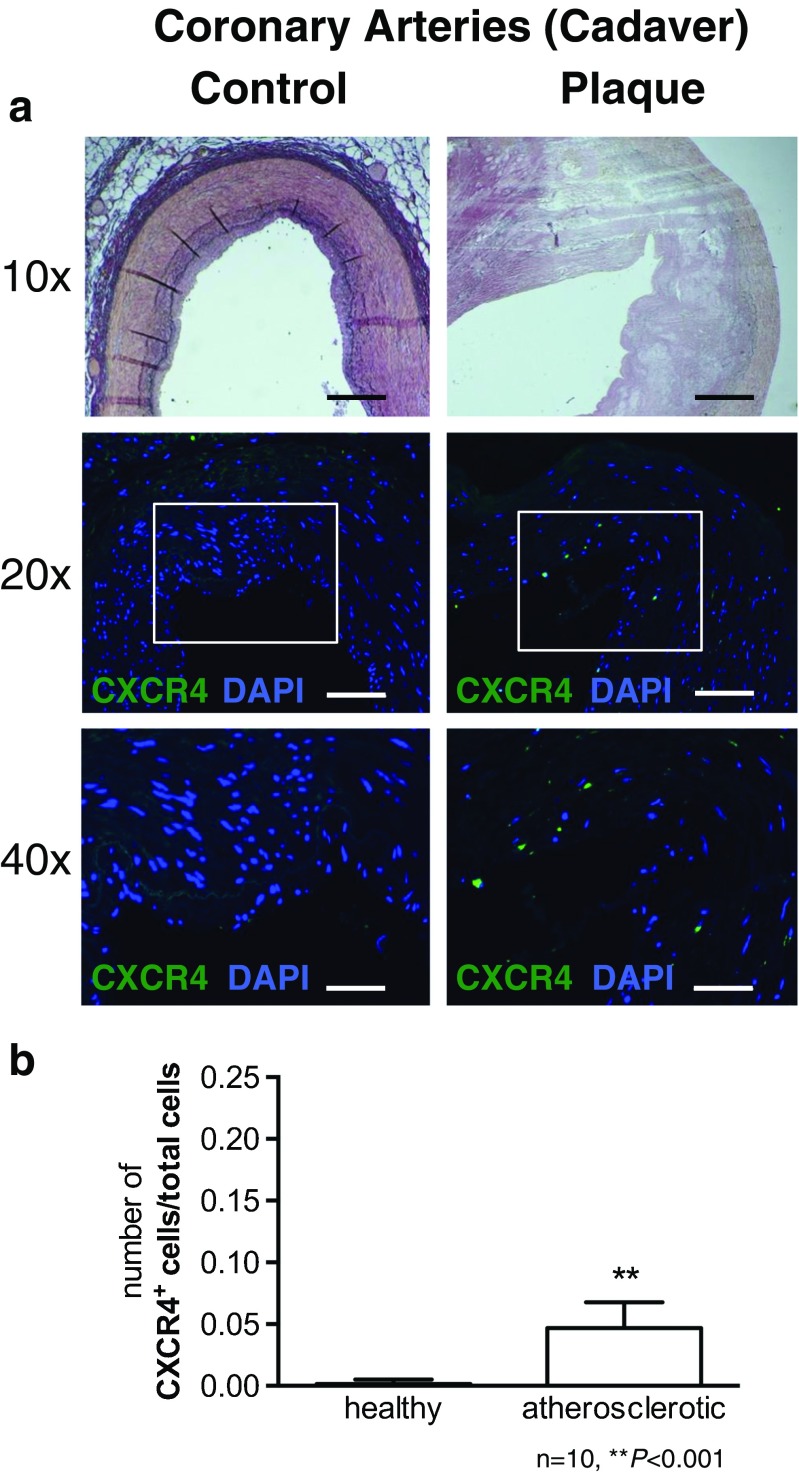
Expression of CXCR4 in human coronary arteries. **a** Immunofluorescence staining of healthy artery wall (*left*) and atherosclerotic artery wall (*right*) reveals a higher number of CXCR4-expressing mononuclear cells (*green; blue* reflects DAPI staining of nuclei). **b** Quantification of CXCR4^+^ cells relative to all cells (scale bars: 10x = 200 µm, 20x = 100 µm, 40x = 50 µm)

**Fig. 2:**
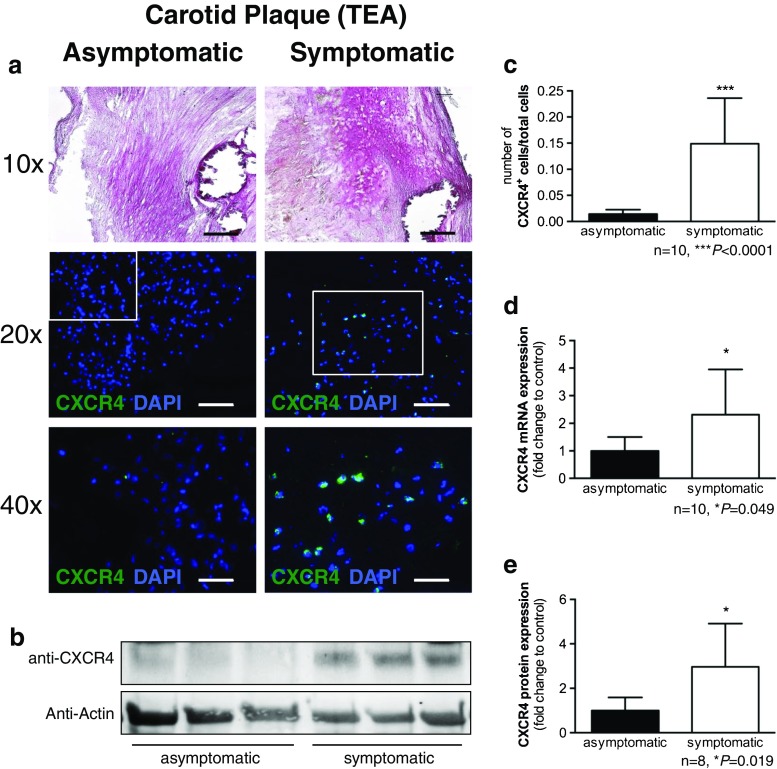
CXCR4 expression in human atherosclerotic carotid plaques. **a** Immunofluorescence staining reveals a higher number of CXCR4-expressing mononuclear cells in plaques of symptomatic patients (*right*) than in plaques of asymptomatic patients (*left*; *green* CXCR4, *blue* DAPI staining of nuclei). **b** Representative western blot shows stronger CXCR4 protein content in samples from symptomatic patients. **c** Quantification of CXCR4^+^ cells relative to all cells on fluorescence microscopy (individual examples shown in **a**). **d** Analysis of CXCR4 mRNA expression as determined by qPCR. **e** Densitometric analysis of western blots in groups (individual blot examples shown in **b**) (scale bars: 10x = 200 µm, 20x = 100 µm, 40x = 50 µm)

**Fig. 3:**
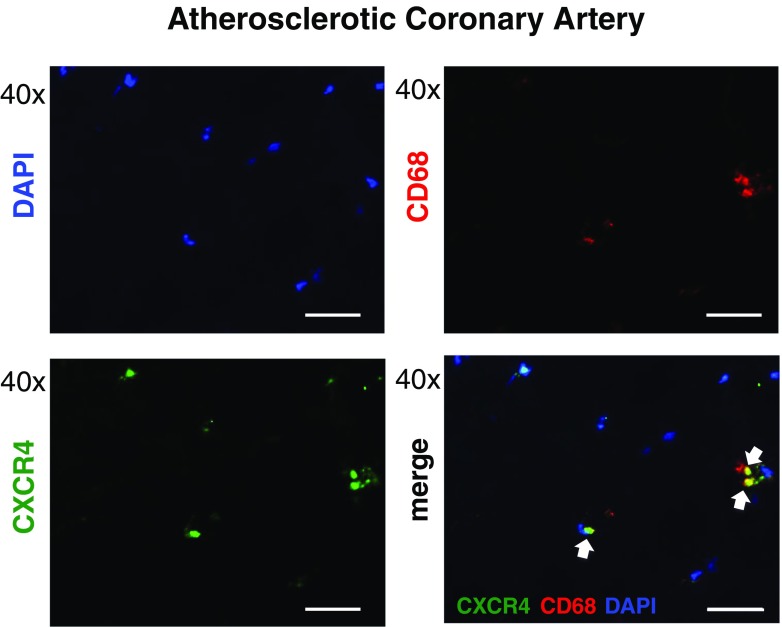
CXCR4 in atherosclerotic plaques of human coronary arteries is predominantly expressed on leucocytes. Immunofluorescent costaining of CXCR4 (*green*, *bottom left*) with the macrophage lineage marker CD68 (*red*, *top right*) and DAPI staining of nuclei (*blue*, *top left*) reveals that the vast majority of CXCR4^+^ cells are CD68^+^ leucocytes (*merged image*, *bottom right*, *arrows*) (scale bars: 40x = 50 µm)

Immunofluorescence revealed an elevated number of CXCR4^+^ cells in human coronary atherosclerotic plaque specimens compared with morphologically healthy coronary artery sections (Fig. 1; *n* = 10 each; *P* < 0.001). Significantly higher numbers of CXCR4^+^ cells were also identified in carotid plaque sections from symptomatic patients than in sections from asymptomatic patients (Fig. 2; *n* = 10 each; *P* < 0.0001), and upregulation of CXCR4 mRNA and protein expression were confirmed by qPCR and western blotting. Dual staining for CXCR4 and the macrophage lineage marker CD68 revealed a high incidence of costaining (Fig. 3). Finally, autoradiography of sections incubated with [^68^Ga]pentixafor demonstrated significantly higher radioactivity in symptomatic lesions than in asymptomatic lesions (26.47 ± 20.82 kBq/mm^3^ vs. 15.77 ± 8.04 kBq/mm^3^; *P* = 0.034).

### In vivo PET/CT identifies CXCR4 signal in culprit stented coronary artery lesions after acute myocardial infarction

**Fig. 4:**
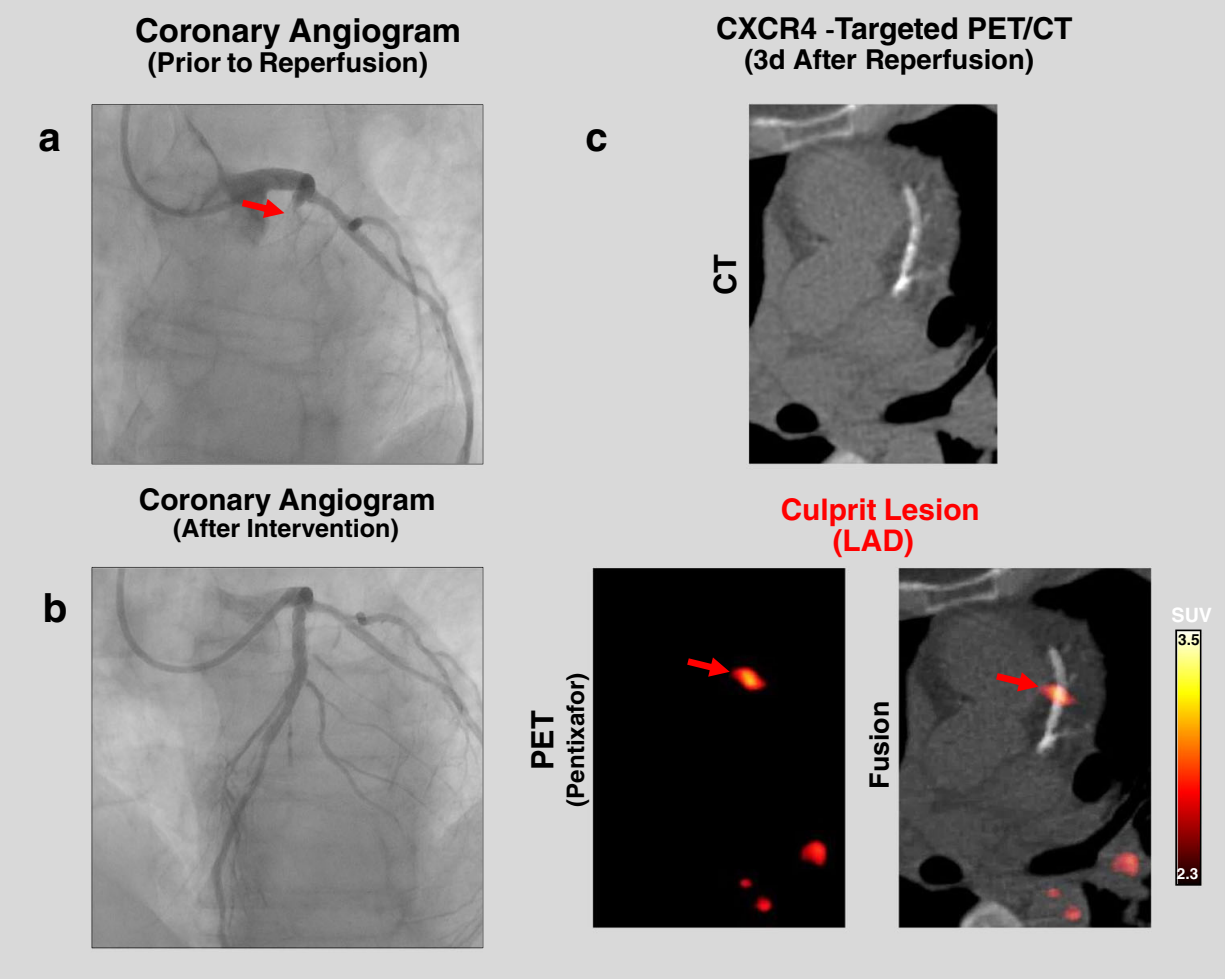
[^68^Ga]Pentixafor PET/CT identifies CXCR4 upregulation in culprit coronary lesions after acute myocardial infarction and stent-based reperfusion. **a** Coronary angiogram prior to reperfusion shows LAD occlusion (*arrow* lesion). **b** Coronary angiogram after stent-based LAD reperfusion. **c** [^68^Ga]Pentixafor PET/CT after reperfusion showing focal CXCR4 signal at the site of the LAD culprit lesion fusing to the stent location (i.e. CXCR4^+^ culprit lesion)

Qualitatively, focal CXCR4 signal was detected by PET in the culprit stented coronary lesions in 32% of patients on ungated images (Fig. 4; Supplementary Fig. 1, image without attenuation correction). Sampling of the five different motion-corrected PET/CT datasets was feasible in all patients, but dual cardiac and respiratory gating could not be performed in three patients due to incomplete data. Motion correction techniques improved lesion detectability in vivo. Detectability of culprit lesions was lowest for ungated images (32% of patients) and highest for dual-gated images (85%, *P* < 0.001), followed by amplitude-based respiratory gated images (81%, *P* < 0.001), cardiac gated images (68%, *P* = 0.005), and list-mode data-driven gated images (54%, *P* = 0.10).

**Fig. 5:**
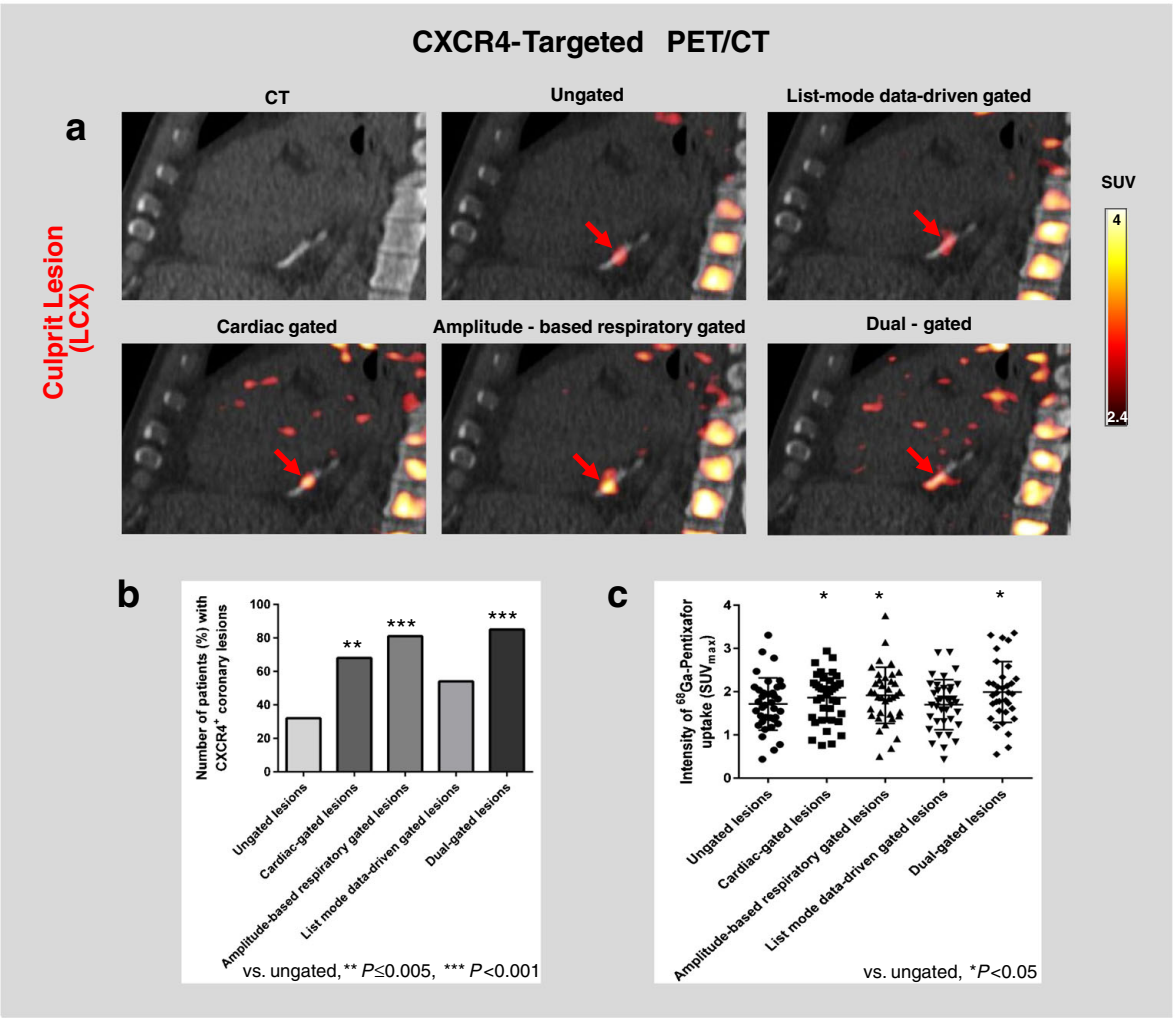
Effect of motion correction on [^68^Ga]pentixafor uptake and detectability in culprit coronary lesions. **a** [^68^Ga]Pentixafor PET/CT images of a culprit LCX lesion. Focal CXCR4 signal (*red*) is more clearly depicted after cardiac, respiratory motion or dual cardiac/respiratory motion correction (*lower row*) compared to ungated images (*upper row*, *middle*). **b** Detection rates for CXCR4^+^ culprit coronary lesions are higher in gated images (*P* ≤ 0.005). **c** Signal intensity of CXCR4^+^ culprit coronary lesions is higher in gated images (*P* < 0.05). Background subtraction was performed using an individually adjusted threshold in this particular subject for clearer visualization of tracer uptake

Quantitatively, using dual gating as the best motion correction technique, the median SUV_max_ of focal CXCR4 signal in the culprit stented coronary lesions was 1.96 (IQR 1.55–2.31). Motion correction techniques improved signal intensity in vivo (*P* = 0.0002; Supplementary Fig. 2). The signal intensity of culprit lesions was lowest on ungated images (median SUV_max_ 1.69, IQR, 1.34–2.08), and was significantly elevated on dual-gated images (mean of differences 0.28), amplitude-based respiratory gated images (mean of differences 0.20), and cardiac gated images (mean of differences 0.15; *P* < 0.05 in all cases), but not list-mode data-driven gated images (mean of differences −0.01; *P* > 0.05). The median blood-pool SUV was 1.8 (IQR 1.7–2.0) on ungated images. Examples and key results are summarized in Fig. 5.

### CXCR4 signal identified by targeted PET/CT is highest in culprit stented coronary lesions

**Fig. 6:**
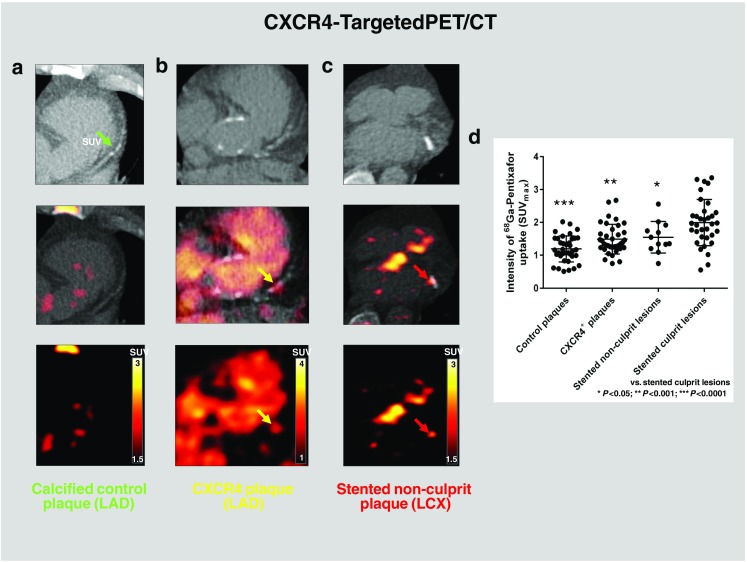
[^68^Ga]Pentixafor PET/CT identifies CXCR4 upregulation in various types of coronary lesions. **a** Calcified LAD plaque without visually identified uptake (control). **b** CXCR4^+^ LAD plaque, partially calcified on CT and with CXCR4 signal. **c** Focal CXCR4 upregulation at the site of a stented LCX nonculprit lesion fusing to the stent location. **d** Signal intensity of CXCR4 expression is highest in stented culprit lesions and lowest in calcified plaques without visually identified uptake (controls) (ANOVA, *P* < 0.0001). The images were obtained from different patients. Background subtraction was performed using individually adjusted thresholds for clearer visualization of tracer uptake

**Fig. 7:**
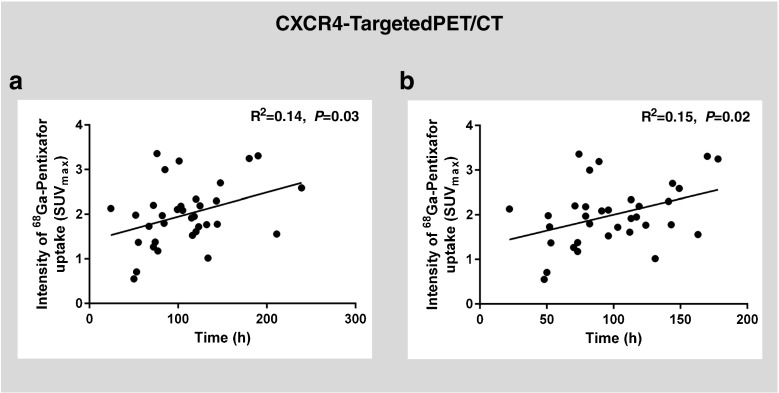
Temporal evolution of [^68^Ga]pentixafor signal. There was a mild but significant correlation between CXCR4 PET signal in culprit lesions and (**a**) time after symptom onset (*R*^2^ = 0.14, *P* = 0.03, and (**b**) time after stenting (*R*^2^ = 0.15, *P* = 0.02; dual-gated data)

Using dual gating, an elevated CXCR4 signal was also detectable in stented nonculprit coronary lesions (*n* = 12), but the intensity was lower than in culprit stented lesions (median SUV_max_ 1.45, IQR 1.23–1.88, vs. 1.96, IQR 1.55–2.31; *P* = 0.048). Also, a group of CXCR4^+^ nonculprit nonstented lesions was identified by a focal CXCR4 hot spot on PET images (*n* = 36). The signal strength in this group, however, was markedly lower than in culprit stented lesions (median SUV_max_ 1.34, IQR 1.23–1.74; *P* = 0.0005). In addition, the signal strength in the control group of calcified nonculprit nonstented lesions was also markedly lower than in culprit stented lesions in the same patient (median SUV_max_ 1.16, IQR 0.98–1.52; *P* < 0.0001; Fig. 6). Consistently, there was a mild but significant correlation between CXCR4 PET signal in culprit lesions and time after stenting (*R*^2^ = 0.15, *P* = 0.02) as well as time after symptom onset (*R*^2^ = 0.14, *P* = 0.03; Fig. 7). Finally, elevated CXCR4 expression was noted in acutely infarcted myocardium in all patients (median SUV_mean_ 2.30, IQR 2.04–2.77). The signal intensity in injured coronary arteries was not associated with CXCR4 expression in acutely infarcted myocardium (*R*^2^ = 0.005, *P* = 0.70), spleen (SUV_mean_ 5.37, IQR 4.88–6.80; *R*^2^ = 0.02, *P* = 0.45), or bone marrow (SUV_mean_ 2.93, IQR 2.53–3.45; *R*^2^ = 0.04, *P* = 0.25).

The CXCR4 signal in the culprit stented lesion was not significantly different between patients with and without statin treatment (median SUV_max_ 1.7, IQR 1.1–2.5, vs. 2.0, IQR 1.6–2.3; *P* = 0.46), and was also not different between patients with and without low-dose acetylsalicylic acid treatment (median SUV_max_ 1.5 , IQR 1.1–2.6, vs. 2.0, IQR 1.6–2.3; *P* = 0.39).

## Discussion

This study supports the feasibility of targeted PET for visualization of CXCR4 expression in coronary artery lesions. It further establishes [^68^Ga]pentixafor as a molecular imaging marker of CXCR4 expression after vessel wall injury. Clinical PET/CT, combined with motion-correction techniques, identified upregulated CXCR4 signal in culprit coronary lesions early after myocardial infarction and stent-based reperfusion. However, it also identified a CXCR4 signal in subgroups of stented nonculprit lesions, and in nonstented nonculprit lesions, suggesting that both native plaque inflammation and stenting-induced vascular injury can be detected and add up to the signal from culprit lesions. These clinical data are supported by work-up of tissue samples ex vivo, which confirmed CXCR4 expression in advanced atherosclerotic plaque and localized it mainly to inflammatory cells.

The CXCL12/CXCR4 axis plays a crucial role in leucocyte trafficking throughout the body [4]. Monocytes and macrophages express CXCR4 and are recruited via gradients of CXCL12 released at sites of tissue injury [26, 27]. The CXCR4/CXCL12 axis also participates in the rapid release of neutrophils from bone marrow during inflammation [28]. Marked infiltration of culprit atherosclerotic lesions by neutrophils in acute coronary syndromes has been reported [29]. Moreover, previous studies have revealed progressive accumulation of CXCR4^+^ cells during plaque progression, with the highest expression in unstable plaque [30]. In addition, vascular injury increases CXCL12 expression in plasma and vessel wall [8, 27], mediated by apoptosis of intimal cells [31]. Importantly, CXCR4 is also expressed by endothelial and smooth muscle cells in the arterial wall [4], indicating that other cell types make a relevant contribution to the in vivo CXCR4 signal [32]. Although the vast majority of CXCR4^+^ cells in the immunofluorescence analyses in this study were CD68^+^ macrophages, we also observed CD68^−^ CXCR4^+^ cells which may represent other CXCR4^+^ inflammatory cells, but also other cells, e.g. vascular smooth muscle cells. However, their precise nature could not be determined using the applied methodology.

Analysis of various subgroups of coronary lesions in the present study suggests that the increased CXCR4 signal in culprit coronary lesions represents a mixture of both inflammation of a vulnerable plaque that led to rupture and subsequent infarction, as well as inflammation in response to later stent-based reperfusion-related vessel wall injury. Consistently, stented nonculprit lesions also demonstrated upregulated CXCR4 signal, albeit significantly lower than in culprit stented lesions. Intriguingly, the motion-corrected imaging approach also identified foci of CXCR4 upregulation in nonstented nonculprit coronary lesions. This signal was again significantly lower than in culprit stented lesions and, given the detection limits of PET/CT, probably represents only the most intensely CXCR4^+^ native coronary regions. However, the findings are consistent with ex vivo demonstration of an increasing number of CXCR4^+^ cells within the vascular wall as atherosclerotic lesions progress, and they are also consistent with prior intravascular ultrasound observations of several high-risk plaque ruptures at sites other than the culprit lesion in acute coronary syndromes [33, 34].

Indeed, recent preclinical and clinical studies have demonstrated that elevated CXCR4 expression can be detected using [^68^Ga]pentixafor PET in most parts of the arterial tree in both histologically stable and unstable plaque [14, 15]. Interestingly, the CXCR4 PET signal in injured coronary plaque tended to increase with time after reperfusion in the early time-frame covered in this study, and it differed from the temporarily elevated CXCR4 signal in damaged myocardium, spleen and bone marrow, which peaks within 3–5 days and then declines as a function of the postinfarct myocardial and systemic inflammatory response [12]. Along with the detectable CXCR4 signal in stented nonculprit lesions, this observation might be a result of differences in the evolution of inflammatory processes in the myocardium and plaque over time, but also suggests a relevant contribution of stent-induced injury to the signal. Future studies including patients before stent-based reperfusion are needed to determine the relative contribution of native plaque inflammation versus stent-induced injury to the measured composite CXCR4 signal. The current study provides proof-of-principle that CXCR4-targeted imaging of CXCR4^+^ coronary plaque is feasible, and it thereby lays the foundation for such future projects. Speculatively, [^68^Ga]pentixafor coronary PET/CT may even be useful for guiding antiinflammatory treatment or even treatment targeting CXCR4. This has been suggested, for example, to reduce neointimal lesion size, smooth muscle progenitor cell mobilization and neointimal proliferation after experimental arterial injury [35], and noninvasive detection of the presence of the target may be helpful for clinical translation of such interventions.

Besides the novel molecular target, our study also included motion correction as a technical innovation in PET that is highly relevant for noninvasive coronary artery imaging. The detection of small lesions on PET is generally complicated by three-dimensional image blurring introduced by the finite spatial resolution of the imaging system and by image sampling on a voxel grid that does not match the actual contours of the tracer distribution [36]. Because PET acquisition is not fast enough to enable breath-hold imaging, respiratory motion along with intrinsic cardiac motion causes further blurring. Together with the limited spatial resolution, this leads to deviation of the apparent signal intensity from true values as a result of partial volume effects. Motion correction can significantly reduce blurring by accounting for both respiration-induced and cardiac movement [20]. Of note, the detectability of culprit coronary lesions, which provided the strongest signal, was still low when using ungated images, but increased significantly when using motion correction. Dual correction of cardiac and respiratory motion yielded the best results. Relatively small differences in SUV_max_ between different gating approaches led to relatively high differences in detection rates. This may be explained by the fact that blood-pool signal is usually relatively high using [^68^Ga]pentixafor PET (SUV_mean_ 1.8, IQR 1.7–2.0) and the lesion to blood-pool signal ratio may be relatively low. Therefore, relatively small increases in signal intensity may lead to markedly higher detection rates (e.g. the mean of differences for dual-gated images compared to ungated images was 0.28). It is also important to keep in mind that localization of culprit lesions was guided by CT (i.e. stents), and not by the PET signal itself. Therefore, knowing precisely which lesion to assess may allow a lesion with even a small increase in tracer intensity to be judged as visually [^68^Ga]pentixafor-positive when the signal exceeds that of the blood pool.

In addition to gating, we also applied point-spread function modelling and time-of-flight reconstruction techniques (Ultra High-Definition, UHD) which have been shown to considerably improve signal intensity for PET analysis [37]. This study established and evaluated a sophisticated dual-gated imaging approach for coronary artery imaging on UHD PET. By contrast, list-mode data-driven gating did not significantly improve lesion detectability. This approach is based directly on frame-by-frame analysis of PET emission data, without the use of external trigger signals [23, 24]. It requires clear contours of the organ to be tracked, such as the myocardium in case of ^18^F-FDG imaging, but such myocardial contours are not visible in [^68^Ga]pentixafor PET images owing to lack of uptake in normal myocardium, which on the other hand is an advantage for delineation of coronary uptake without spillover.

Some limitations of the present study should be acknowledged. Obviously, not all lesions observed on PET in vivo can be verified by histology (which applies to all clinical atherosclerosis studies). However, there are recent clinical studies evaluating the usefulness of pentixafor for CXCR4 imaging in plaque [14, 38], and also studies which have demonstrated that the arterial wall uptake can be blocked, is specific, reproducible, and correlates with the presence of CXCR4^+^ cells [16, 38]. In addition, the ex vivo data presented in this work confirm the presence of CXCR4^+^ cells in the coronary wall, and demonstrate an increasing number of these cells with advancing pathology of lesions, in line with the observed in vivo PET data. Second, the signal might have been enhanced by attenuation correction related to the proximity of a metallic stent. However, the signal seen in stented culprit lesions was also seen on images without -attenuation correction, and was significantly higher than in stented nonculprit lesions, supporting the basic hypothesis of this work, and confirming that the stent signal was not caused by attenuation correction. The precise cell population contributing to the in vivo [^68^Ga]pentixafor signal could not be identified. However, ex vivo data showed upregulated CXCR4 expression in atherosclerotic coronary arteries and/or in carotid arteries from symptomatic subjects. This finding was confirmed by mRNA expression, protein expression and immunofluorescence. Moreover, uptake of [^68^Ga]pentixafor was validated by ex vivo autoradiography in carotid arteries, and immunofluorescence dual-staining of CD68 confirmed CXCR4 expression by leucocytes. In addition, two recent experimental studies investigating carotid arteries [15, 16] have shown that CXCR4 expression in injured plaque is predominantly found on leucocytes, supporting our conclusions.

### Conclusion

In this proof-of-principle study, we demonstrated the feasibility of motion-corrected targeted PET imaging with [^68^Ga]pentixafor for identifying increased CXCR4 expression in injured culprit and nonculprit coronary artery plaques triggered by vessel wall inflammation, but also by stent-induced injury. This novel noninvasive approach may refine the clinical characterization of atherosclerotic lesions, serving as a platform for novel diagnostic and therapeutic approaches targeting coronary atherosclerotic plaque biology.

## Electronic supplementary material

ESM 1 (DOC 47 kb)

ESM 2 (PPT 1396 kb)

ESM 3 (PPT 1131 kb)
