# Supplementary material for: Imaging of chemokine receptor CXCR4 expression in culprit and nonculprit coronary atherosclerotic plaque using motion-corrected [68Ga]pentixafor PET/CT
Source: Eur J Nucl Med Mol Imaging. 2018 Jul 3;45(11):1934–44. doi: 10.1007/s00259-018-4076-2 (PMC6132552; doi:10.1007/s00259-018-4076-2)
Supplement: Supplementary file 1 — (DOC 47 kb) [file 259_2018_4076_MOESM1_ESM.doc]

**Imaging of chemokine receptor CXCR4 expression in culprit and non-culprit coronary atherosclerotic plaque using motion-corrected [^68^Ga]Pentixafor PET/CT**

Thorsten Derlin, MD^1^*; Daniel G. Sedding, MD^2^; Jochen Dutzmann, MD^2^; Arash Haghikia, MD^2^; Tobias König, MD^2^; L. Christian Napp, MD^2^; Christian Schütze, MSc^1^; Nicole Owsianski-Hille^1^; Hans-Jürgen Wester, PhD^4^; Saskia Kropf^5^, James T. Thackeray, PhD^1^; Jens P. Bankstahl, PhD^1^; Lilli Geworski, PhD^3^; Tobias L. Ross, PhD^1^; Johann Bauersachs, MD, FESC, FAHA^2^; and Frank M. Bengel, MD, FAHA^1^

^1^Department of Nuclear Medicine,

^2^Department of Cardiology and Angiology,

^3^Department of Radiation Protection and Medical Physics,

Hannover Medical School, Hannover, Germany

^4^Radiopharmaceutical Chemistry, Technical University of Munich, Germany

^5^Scintomics GmbH, Fürstenfeldbruck, Germany.

***Address for correspondence:**

Thorsten Derlin, MD

Department of Nuclear Medicine

Hannover Medical School

Carl-Neuberg-Str. 1

D-30625 Hannover, Germany

Tel.: +49 (0) 511 532 2577

Fax: +49 (0) 511 532 3761

E-mail: [Derlin.Thorsten@mh-hannover.de](mailto:Derlin.Thorsten@mh-hannover.de)

***ONLINE SUPPLEMENT***

***Ex Vivo* Tissue Analysis**

*Tissue samples.* Cadaveric coronary artery specimens with or without atherosclerotic lesions (n=10 each) were obtained from ProVitro (Berlin, Germany). Additionally, carotid plaque specimens from patients with symptomatic (transient ischemic attack or stroke in the previous <=30 days; n=10) or asymptomatic severe (>80%) carotid stenosis (n=10), who had undergone elective carotid thrombendatherectomy for repair, were analyzed. Samples were embedded in Tissue Tek OCT embedding medium (Sakura Finetek Europe B.V., Zoeterwoude, The Netherlands). Afterwards, the arteries were snap-frozen and stored at -80°C until sectioning and further analysis.

*Immunofluorescence.* Cross sections (6μm thick) were mounted on slides treated with 0.1% poly-l-lysine solution (Sigma-Aldrich, St. Louis, MO, USA). Antigen retrieval was performed using 10mM citrate buffer in a pressure cooker (7min) or in CXCR4-specific target retrieval buffer (S2367, pH 9.0, Dako REALTM, Inc) using a microwave oven (20min). Subsequently, sections were pre-treated with 1% bovine serum albumin in background reducing diluents (S2022, Dako REAL, Inc), and incubated with anti-CXCR4 antibody (ab124824, Abcam Inc.; dilution 1:200) and/or with an anti-CD68 antibody (ab955, Abcam Inc.; dilution 1:200). Thereafter, samples were incubated with Alexa 488-conjugated secondary antibodies (LifeTechnologies, Carlsbad, CA, USA) and counterstained with nuclear 4.6-diamidino-2-phenylindole (Immunoselect Antifading Mounting Medium DAPI, Dianova GmbH, Hamburg, Germany). For negative control, primary antibody was substituted with species- and isotype-matched control antibody (Santa Cruz Biotechnology Inc., Dallas, TX, USA).

*Microscopy.* Tissue samples were analyzed using bright field and immunofluorescence microscopy (Eclipse TE2000-S, Nikon Instruments Europe B.V., Amstelveen, The Netherlands) equipped with filter blocks and image processing software.

*Quantitative real-time RT-PCR.* In carotid plaques, isolation of total RNA and synthesis of cDNA were performed using commercial kits (RNeasy Mini Kit, Qiagen GmbH, Hilden, Germany and High Capacity RNA-to-cDNA-Kit, Applied Biosystems, Foster City, CA, USA). Real-time PCR was performed on a Bio-Rad CFX96 Touch™ Real-Time PCR Detection System using the iQ™ SYBR® Green Supermix (Bio-Rad Laboratories Inc., Hercules, CA, USA). Primers were as follows: C-X-C motif chemokine receptor 4 (CXCR4) forward 5’-ACCCCATCCTCTATGCTTTCCT-3’, CXCR4 reverse 5’-ATGTCCACCTCGCTTTCCTTT-3’, eukaryotic translation initiation factor 3 subunit D (EIF3D) forward 5’-ATCCTGCCTAAGAGTGCCAA-3’, EIF3D reverse 5’-CTCGGGGTTTCTGTGATTTCTG-3’, phosphoribosyl pyrophosphate synthetase-associated protein 1 (PRPSAP1) forward 5’-TCCCCGCCTATGGTCAAAAA-3’, PRPSAP1 reverse 5’-GCCTCCAACATCTCCAACTAC-3’, solute carrier family 7 member 6 opposite strand (SLC7A6OS) forward 5’-GAGGAGGAAAGAGGCAGCA-3’, SLC7A6OS reverse 5’-GGACATCACAAGACCATCAGT-3’, zinc finger protein 394 (ZNF394) forward 5’-GCCCCCTGTTTTCTAAGTGTG-3’, ZNF394 reverse 5’-ATGCTGTTGAGTCCTTCTGCT-3’. All primers were designed using NCBI Primer-BLAST. Oligos were analyzed for potential self- and hetero-dimer formation using OligoAnalyzer 3.1. Primers and expected PCR products were tested for hairpins and purchased from Eurofins MWG Operon LLC (Huntsville, AL, USA). EIF3D, PRPSAP1, SLC7A6OS, and ZNF394 were selected as potential reference genes using Genevestigator RefGenes (Nebion AG, Zurich; Switzerland). Expression stability was measured for all selected reference genes using the geNorm algorithm. To quantify changes in gene expression, the 2^-ΔΔCt^ method was used to calculate relative fold changes normalized to the geometric mean of the three reference genes with the highest expression stability. All analyses were performed in triplicate, and either the DNA template or the reverse transcriptase was omitted for control reactions.

*Immunoblot analysis***.** For western blotting from carotid plaque, samples were homogenized in a glass homogenizer in 300 μL ice cold radioimmunoprecipitation assay (RIPA) buffer. After centrifugation for 20 min at 12.000 rpm at 4°C, the supernatants were frozen at -80°C until further analysis. The cleared supernatants from tissue lysates were run on 4-12% Bis-Tris gels (NuPAGE® SDS-PAGE Gel System, LifeTechnologies, Carlsbad, CA, USA) and transferred onto nitrocellulose membrane (Trans-Blot®, Bio-Rad Laboratories Inc., Hercules, CA, USA). After blocking, blots were incubated with the primary antibody (CXCR4, ab124824, Abcam Inc.; dilution 1:200 and β-Actin, #4967, Cell Signaling, dilution 1:2000) overnight at 4° C. Proteins were then detected by enhanced chemiluminescence (Pierce ECL Plus, Thermo Scientific, Rockford, IL, USA) after labeling with horseradish peroxidase-labeled secondary antibody (sc-2056 or sc-2004, Santa Cruz Biotechnology Inc., Dallax, TX, USA).

*Autoradiography.* Three sections from carotid plaques were incubated in 40 kBq/mL ^68^Ga-Pentixafor in phosphate buffered saline (PBS) for 30min at room temperature. Slides were then washed sequentially in ice cold PBS and distilled water, dried, and exposed for 60min to a phosphor imaging screen. A standard curve of known activity concentration (0-500 kBq/mL) was prepared and 1 µL aliquots were spotted on thin layer chromatography plate and exposed concurrently. The standard curve was used to calculate activity concentration for each sample, using an ROI covering the full circumference of the vessel in each section, and averaged over sections for each patient slide. Average activity concentration for each patient group was calculated based on a section-by-section quantification.

**Supplementary Figures**

**Supplementary Figure 1** [^68^Ga]Pentixafor PET/CT identifies CXCR4 up-regulation in culprit coronary lesions after acute myocardial infarction and stent-based reperfusion. **A**, CT showing LAD stent. **B**, Dual-gated, attenuation-corrected fused PET/CT and **C**, PET showing focal CXCR4 signal at the site of the LAD culprit lesion, fusing to stent localization (i.e., CXCR4^+^ culprit lesion). **D**, Ungated, non-attenuation-corrected fused PET/CT and **E**, PET confirming focal CXCR4 signal at the site of the LAD culprit lesion, fusing to stent localization (i.e., CXCR4^+^ culprit lesion).

**Supplementary Figure 2** [^68^Ga]Pentixafor PET/CT identifies CXCR4 up-regulation in culprit coronary lesions after acute myocardial infarction and stent-based reperfusion. **A**, CT showing RCA stent. **B**, Ungated, attenuation-corrected PET and **C**, fused PET/CT with no significant uptake at the site of the RCA culprit lesion (SUV_max_, 1.88). **D**, Dual-gated, attenuation-corrected PET and **E**, fused PET/CT showing marked uptake at the site of the RCA culprit lesion (SUV_max_, 2.26). Dual-gating improved uptake and lesion detectability in this patient.
