# Supplementary figures and images for: Imaging of chemokine receptor CXCR4 expression in culprit and nonculprit coronary atherosclerotic plaque using motion-corrected [68Ga]pentixafor PET/CT
Source: Eur J Nucl Med Mol Imaging. 2018 Jul 3;45(11):1934–44. doi: 10.1007/s00259-018-4076-2 (PMC6132552; doi:10.1007/s00259-018-4076-2)

## Slide 1
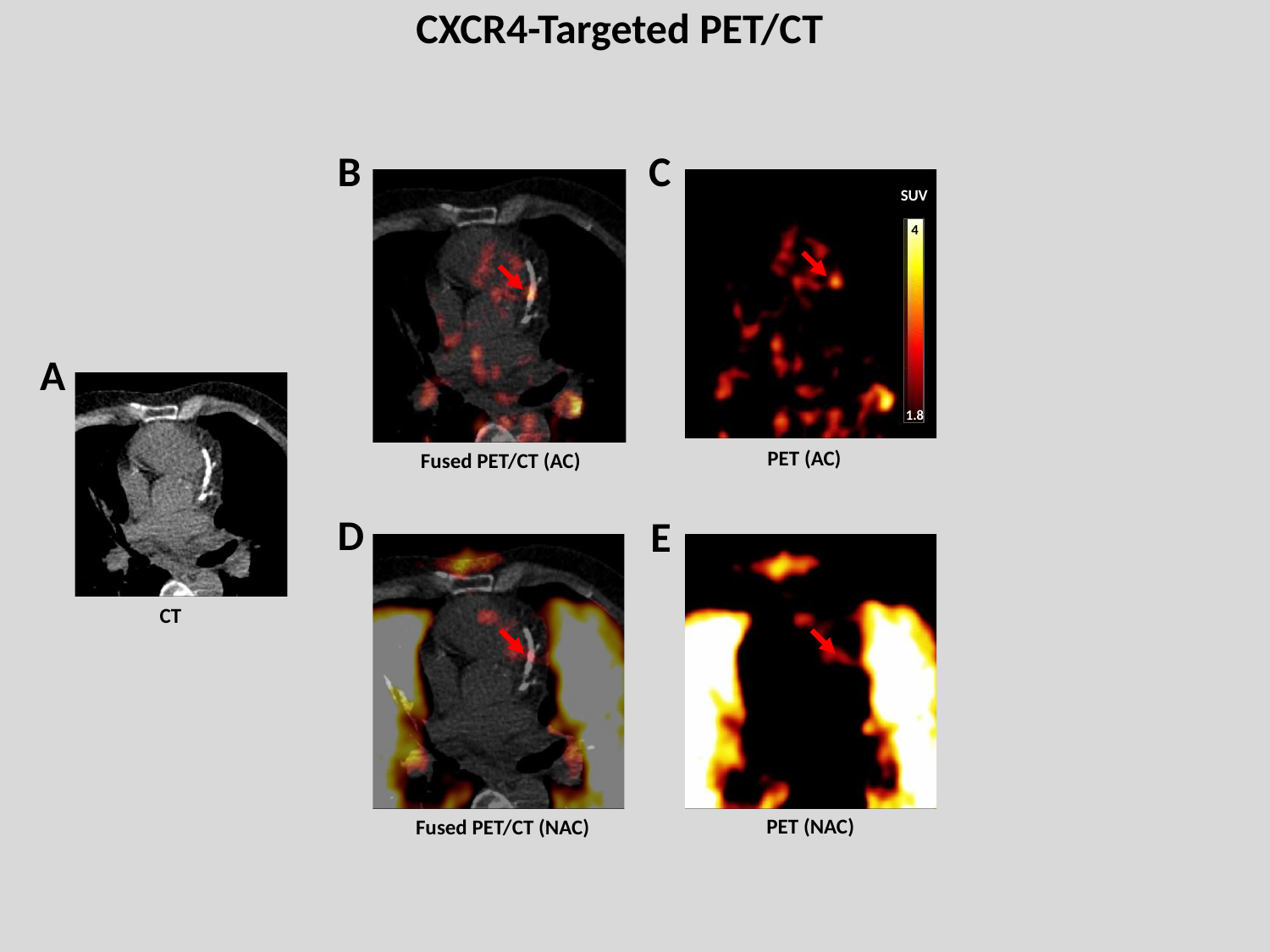

CXCR4-Targeted PET/CT
B
C
SUV
4
A
1.8
PET (AC)
Fused PET/CT (AC)
D
E
CT
PET (NAC)
Fused PET/CT (NAC)

Supplement: Supplementary file 2 — (PPT 1396 kb) [file 259_2018_4076_MOESM2_ESM.ppt]

## Slide 1
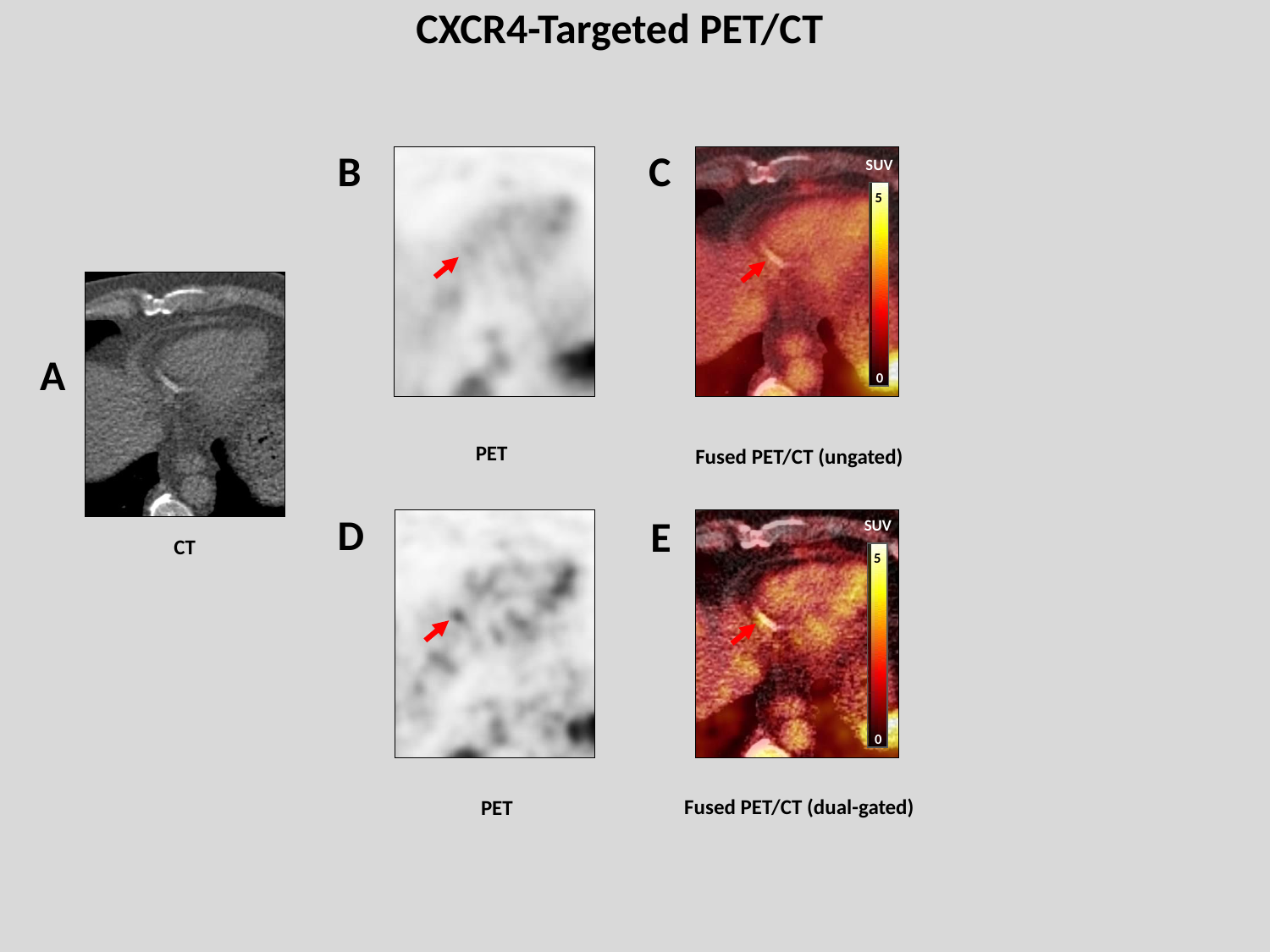

CXCR4-Targeted PET/CT
B
C
SUV
5
A
0
PET
Fused PET/CT (ungated)
D
E
SUV
CT
5
0
Fused PET/CT (dual-gated)
PET

Supplement: Supplementary file 3 — (PPT 1131 kb) [file 259_2018_4076_MOESM3_ESM.ppt]
